# Beliefs, self-destruction, and the rational mind

**DOI:** 10.3389/fpsyg.2014.01231

**Published:** 2014-11-07

**Authors:** Claire M. Fletcher-Flinn

**Affiliations:** College of Education, University of OtagoDunedin, New Zealand

**Keywords:** human rationality, evolutionary theory, self-destructive beliefs, experimental philosophy, action-belief systems


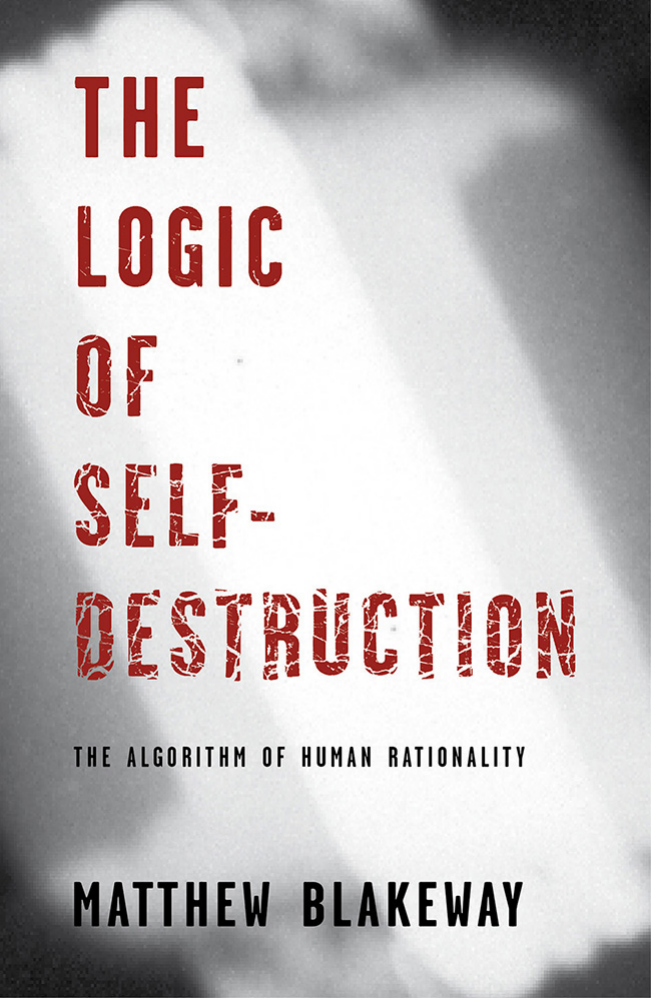


This is a book, both comprehensive and thought-provoking, with a timely thesis that is a “must read” for anyone interested in human behavior and our collective future. Matthew Blakeway takes the view that our brains are biological computers with algorithms for action. But what if the algorithms are based on faulty data? What if our actions, based on our beliefs, are a product of causal circularity?

Blakeway reminds us that we cannot directly observe people's emotions and beliefs. They must be induced from behavior–what we say and do. He contends that humans are biologically driven to create environments that enhance our survival by creating favorable emotional outcomes, and avoiding those that are less advantageous. Emotions are the drivers of our actions, but he proposes that they can become corrupted through tactical deception. This corruption happens when we logically derive our understanding of emotions from the affected or suppressed behavior of others (or ourselves). We deduce an incorrect understanding because the input is invalid. This results in false beliefs of which we are unaware, but they affect our own behavior. Language allows us to think and re-visit our emotional responses, anchoring them further in memory. Our thoughts and beliefs become irrefutable logical tautologies. This results in actions for ourselves and our societies that are no longer fitness-enhancing, but are self-destructive.

Blakeway makes an important distinction between statements of “believing *in*” and “believing *that*.” The latter can be subjected to scientific scrutiny and experimentation, but the former cannot, as a belief *in* is irrefutable. Presumed true because they cannot be falsified, having beliefs *in* makes human conflict inevitable. History is replete with examples of incompatible belief systems that drive conflict and war, the latest being the mass displacement and slaughter of thousands of people in Iraq by the Islamic State. Furthermore, the actual words that are used to talk about ideological concepts, like “democracy” or “capitalism,” encapsulate compound beliefs. The more accumulated concepts that your understanding of the ideological term has acquired, the more prone you (and others) are to “belief drift” and conceptual thought-fluff.

Making this belief distinction also explains why it is so difficult to change strongly held views with the presentation of scientific evidence that contradicts them. Blakeway argues that ideology always takes precedence over empirical evidence because ideology is a trap based upon a causal circularity that leads to a tautological understanding; it is simply and unquestionably considered true from the perspective of its believers. Its own truth is created through a belief feedback loop—an inherent “wheel of doom.”

Ideologies can be hoodwinked by circumventing their original intent. For example, the pursuit of arbitrage by capitalists was of benefit to society, but now leads to both a reallocation, and at the same time, a diminution of wealth through the use of hedge funds, and other economic manipulations. In other circumstances, rather than acknowledge that a belief is untenable and even destructive to our wellbeing as seen by accumulating evidence, a tipping point is reached and we quite rationally impute that it is *we* who failed the belief, and the belief itself remains intact. These failed beliefs can turn into “purer” and more extreme forms of the same belief. At a societal level, this leads to the spiraling pursuit of ideological fundamentalism that has a logical contradiction at its core. Blakeway provides good examples of this with regard to the current surge of both political and religious fundamentalism in the world today.

The description of the process by which our emotions become distorted is plausible. It hinges on evolutionary theory and socialization. Although Blakeway doesn't mention human development to any large degree, the evidence (for a review, Astington, 1994) does support his view. From the beginning babies prefer social to nonsocial stimuli, and by 5 months they are able to distinguish different expressions of emotion. Social interaction is the meeting of minds, and by 2 years, children understand and talk about their mental states, including beliefs, desires, and intentions. A year later they take on the minds of others in their pretend play, and by 4 years, children can recognize, and create false beliefs in others through deliberate acts of deception. Similarly, as early as the first few months, there is social pressure to suppress negative emotion, and “emotional masks” are evident in 3-year-olds (Kieras et al., 2005). We are hard wired through evolution for understanding (and manipulating) others' minds, and suppressing our emotions.

Blakeway moves the evolutionary argument one step further. Anger and revenge as a response to humiliation has an evolutionary basis with regard to parental investment and sexual selection. The revenge response maximizes male biological fitness by removing threats to gene transmission. He contends that if acts of humiliation, by individuals or the state, are met with denial and the suppression of revenge, then the anger that follows these acts becomes misdirected, violent, and targetless. The cause of the destruction is mythologized because we are oblivious to its real source, and revenge escalates in often, unpredictable ways. Ideas of “brain washing,” and “radicalization” are given new explanations, raising the possibility of more effective interventions.

The epilog is entitled “Science and Philosophy Reunite.” The method is a series of thought experiments, and Blakeway's contribution is a new theory of how a human algorithm works to produce behavior. He presents a cohesive argument about the necessity to understand and become aware of our thought traps, and move beyond them to create a world where all can live, without humiliation, anger and violence. Our moral emotions (e.g., pity, sympathy) are also a product of evolution by natural selection, and there is a need to avoid their corruption or suppression, which may become habitual, and as such, contribute to ideological belief-action tautologies. In Darwin's theory, there is only evolutionary press to survive, and as self-referencing biological machines, it is suggested that meaning and purpose in life may best be found by changing our behavior to enhance our emotional outcomes and avoid self-destructive logical actions.

## Conflict of interest statement

The author declares that the research was conducted in the absence of any commercial or financial relationships that could be construed as a potential conflict of interest.
